# Supplementary material for: High proportion of RR-TB and mutations conferring RR outside of the RRDR of the rpoB gene detected in GeneXpert MTB/RIF assay positive pulmonary tuberculosis cases, in Addis Ababa, Ethiopia
Source: PLoS One. 2022 Dec 30;17(12):e0277145. doi: 10.1371/journal.pone.0277145 (PMC9803184; doi:10.1371/journal.pone.0277145)
Supplement: Attachment — Submitted filename: Response to Reviewers.docx [file pone.0277145.s002.docx]

**Dear Dr. Frederic Quinn**

**Academic Editor**

**PLOS ONE**

**PONE-D-22-28840**

High proportion of RR-TB and mutations conferring RR outside of the RRDR of the rpoB gene detected in GeneXpert MTB/RIF assay positive pulmonary tuberculosis cases in Addis Ababa, Ethiopia

PLOS ONE

**Dear Dr. Frederic Quinn;**

Thank you for your comments and for inviting us to submit a revised version.

A. **Comments by Academic Editor:**
1. Comment on Journal Requirements:

- Thank you again and comments related to PLOS ONE’s style requirements including file naming are substantially accommodated.

2. Comment on Data Availability:

- Thank you, and comments are accommodated.
- Our data are not under any of the legal or ethical restrictions and we wanted to share the anonymized metadata set as supporting information, “S1 File-Sociodemographic and GeneXpert assay result Xls” to support the validation of this study. Accordingly, we kindly request your editorial office update the **“Data Availability”** statement we provided during the first submission.

3. Ethical Approval Statement:

- Thank you for your comment. The comment is well accommodated just by moving it to the material and methods section, with content details on IRB, and informed consent.

4. We take note of your comment regarding references and we validated it.

- So far, we do not cite any retracted papers.

All comments from reviewers are accommodated point by point and included in the ‘Response to Reviewers’ file.

**Kindest Regards,**

**Gizachew Taddesse Akalu**

**Corresponding Author**

B. **Response to Reviewer**

| **Serial No.** | **Comment** | **Response** |
| --- | --- | --- |
| **Reviewer #1** | | |
| 1 | Introduction  Ethiopia is among the 30 high TB burden countries with an estimated annual TB incidence rate of 132 per 100 000. The number of people infected with TB is 151 000, 9 900 HIV positive TB incidence, 19 000 HIV negative TB mortality, 2 500 HIV positive TB mortality, 591 individuals diagnosed with DRTB, and 7 cases of laboratory-confirmed pre-XDR TB or XDR TB [9]. **Include the years to which this data is referring.** | Thank you, the comment is accommodated.   - The global TB report in 2020 |
| 2 | Ethical approval Can authors include the ethical **approval number and date** when it was granted? | Thank you. Both comments are accommodated.   - Protocol No. IRB/039/2019 - Date: April 04, .2019 |
| 3 | Inclusion and Exclusion Criteria The inclusion criteria were clinical suspicion of new presumptive pulmonary TB strong enough to warrant a direct sputum investigation and a positive result confirmed by Gene Xpert MTM/RIF. **Take note of the inconsistency in writing the name of the GeneXpert MTB/RIF assay throughout the manuscript.** | Thank you. Comment accommodated and consistency maintained.   - The phrase ‘GeneXpert MTB/RIF assay’ will be maintained and used accordingly. |
| 4 | GeneXpert MTB/RIF Assay Briefly, sputum samples were subjected to processing by approximately mixing with 8ml of GeneXpert sample reagent (SR) containing NaOH [5 - 10%] and isopropyl alcohol [10 – 20%] into the sputum container in a ratio of 2:1 V.V. The mixture was vigorously mixed by shaking the tubes 20 times and incubated for 15 minutes at 20–30 °C. This step has been repeated in between the incubation time. **Take note of the sentence tense used here.** | Thank you. The comment is well accommodated.   - This step was repeated in between the incubation time. |
| 5 | GeneXpert MTB/RIF Assay Performance Characteristics and Result Interpretation Generally, results from GeneXpert MTB/RIF Assay were obtained and assessed with three result types: MTB Detected, MTB Not Detected, and Invalid/Error/No result. **What of MTB was detected with Rifampicin resistance (MTB/RR TB)?** | Thank you. Comment accommodated.   - Yes, MTB Detected RR Detected, MTB Detected RR not Detected and MTB not Detected. |
| 6 | Quality Assurance The internal quality control of the GeneXpert MTB/RIF Assay System was validated using *M. tuberculosis* H37Rv, a non-RIF resistant and known RIF resistant strain stored at -20^o^C. **Which strain is this and how often was this done?** | Thank you. Comment accommodated.   - Laboratory reference strain. Each test does have an inbuilt internal quality assurance system. - When we start a new kit _Batch. |
| 7 | Ethical Considerations Institutional Review Board approval has also been obtained from the Research and Ethics Review Committee of Saint Peter TB Specialized Hospital for the commencement of the study. **Take note of the sentence tense used here.** | Thank you. Comment accommodated.   - Permission was obtained from Saint Peter TB Specialized Hospital before the commencement of the study. |
| 8 | Sociodemographic Characteristics of Study Participants Overall, a total of 216 participants who had enrolled in this study were considered persons with presumptive TB upon clinical suspicion and bacteriologically confirmed with GeneXpert MTB/RIF Assay. **Does Xpert detect the presence of bacteria or bacterial DNA?** | Thank you. We take notes about it.   - Yes, it actually detects bacterial DNA. However, the national guideline defines that: **a bacteriologically confirmed TB case refers to a patient from whom at least one biological specimen is positive for mycobacterium TB by either smear microscopy, Xpert MTB/RIF assay, culture, or other WHO-approved bacteriologic detection tests.** |
| **Reviewer #2** | | |
| I find this piece of work very interesting and useful as I myself did a similar work in Nepal samples looking at the Rifampicin resistance profile. I have to admit that though we saw mutations outside the RRDR, we did not pursue it further, but we always thought that these profiles will be important for enhancing the DNA probes in the future. So, it is an important piece of work.  These are my recommendations. | | Thank you for your excellent contribution, and we are sure that we have benefited from your findings. |
| 1 | I would like to see more clarity on the study design especially pertaining to data related to Rifampicin resistance outside the RRDR. | Thank you.   - We intentionally observed, captured, and categorized data obtained from the GeneXpert assay to further perform probe analysis to assess the utility of each probe within the RRDR of the rpoB gene. - When MTB detected/RR detected, we further looked into which probe was missed or became negative and warrants to understand the probe utility analysis. |
| 2 | I would like to see more clarity in Figure 1, Figure 2, and Table 3. | Thank you for these comments demanding more clarity.   - Figure 1 simply shows a flowchart how to hither, to systematically categorize in a suitable data set to assess and perform probe analysis. - Regarding Figure 2, as results from the GeneXpert assay could be elaborated **semiquantitatively**, we wanted to see the DNA amount in percentage and frequency. - Table 3 is to more elaborate the proportion of RR among missed probes and RR detected without missed probes despite the QC being acceptable. |
| 3 | What was the mutation detected outside RRDR? How was it confirmed as a missense mutation? How was it detected? Do we have any experimental data? I could not find any concrete evidence. | Thank you for this key comment.   - This was obtained from probe analysis results. We only categorically classified the mutation results obtained from the GeneXpert MTB/RIF assay in each probe. - Mutations outside the RRDR have rarely been reported and we observed mutations without missed probe types, i.e., without a negative probe, and we considered these findings outside of the RRDR core region. - Further, we mentioned as a limitation of this study just at the end of the discussion that phenotypic drug susceptibility and target sequence results of rpoB gene are not available at this time. |
| 4 | Do you have any data about any co-mutations on RRDR along with the RR mutation outside this region? | Thank you.   - **No** combination of or multiple missed probe types was observed within the RRDR rpoB gene in this study. - And we reflected this finding on page 12 under the subtitle **“The proportion of RR-TB among missed probes types and RR-TB evolving to escape from RRDR of 81 bp rpoB gene”** |
